# Preparation and fire behavior of rigid polyurethane foams synthesized from modified urea–melamine–formaldehyde resins

**DOI:** 10.1039/c8ra01846d

**Published:** 2018-05-16

**Authors:** Heng Zhu, Shi-ai Xu

**Affiliations:** Shanghai Key Laboratory of Advanced Polymeric Materials, Key Laboratory for Ultrafine Materials of Ministry of Education, School of Materials Science and Engineering, East China University of Science and Technology Shanghai 200237 China saxu@ecust.edu.cn +86-021-64253353; School of Chemical Engineering, Qinghai University Xining 810016 China

## Abstract

In this study, a series of ethylene glycol modified urea–melamine–formaldehyde resins (EUMFs) were synthesized from urea, melamine, paraformaldehyde and ethylene glycol, and then incorporated into rigid polyurethane foams (RPUFs) as a reactive-type liquid flame retardant. The structure of EUMFs was characterized by Fourier transform infrared spectrometry; the morphology of the foams was characterized by scanning electron microscopy; and the thermal degradation and fire behavior of RPUFs were characterized by limiting oxygen index (LOI), cone calorimetry test and thermogravimetry analysis. The results show that the incorporation of EUMFs results in an increase in thermal stability, smoke suppression and LOI of RPUFs. As the melamine loading in EUMFs increases, the peak heat release rate and the total heat release of RPUFs decrease significantly, but the LOI increases slightly. Compared with the original foam, the cells of RPUFs become less regular with nonuniform diameters. In general, EUMFs show excellent flame retardancy and smoke suppression for RPUFs.

## Abstract 2

In this study, a series of ethylene glycol modified urea–melamine–formaldehyde resins were synthesized, and then incorporated into rigid polyurethane foams as a reactive-type liquid flame retardant.

## Graphical abstract

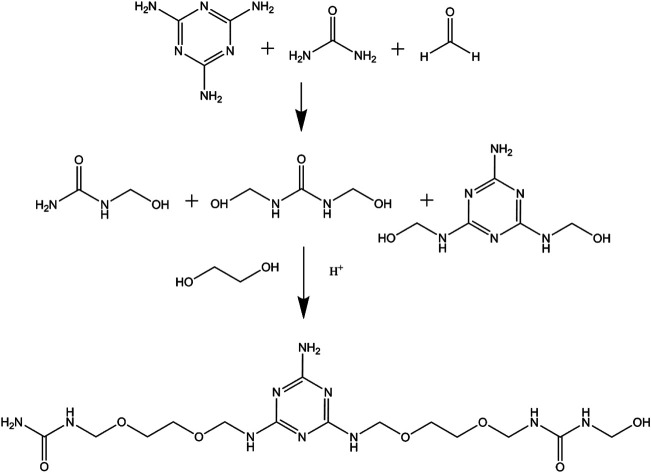

## 1. Introduction

Rigid polyurethane foams (RPUFs) have been used in many fields due to their outstanding insulation performance, dimensional stability, and low density.^1–4^ However, RPUFs are flammable because of the weak covalent bond and foam structure, and notoriously, a large amount of smoke and toxic gases are released during the combustion of RPUFs, which can have severe environmental and health consequences.^5–7^ Obviously, there is a need to improve the flame resistance of RPUFs.

Halogen flame retardants, such as tris(2-chloroethyl)phosphate and tris(1-chloro-2-propyl)phosphate, are mostly used in RPUFs, but their applications are limited due to the release of a large amount of toxic gases during the combustion.^8^ Thus, much effort has been made to develop efficient and eco-friendly halogen-free flame retardants for RPUFs, which can be roughly divided into addition-type and reactive-type. The addition-type flame retardants, such as aluminium hydroxide, expandable graphite, and ammonium polyphosphate, are mainly incorporated into the foam by means of physical mixture.^5,9–13^ However, the mechanical properties of RPUFs can be greatly reduced and the flame retardants would separate from the foam, due to the poor compatibility between flame retardants and foam matrix. The reactive-type flame retardants are organic compounds containing flame retardant elements, such as phosphorus, nitrogen and active functional groups, and they have more durable flame retardant performance and better compatibility with the foam matrix compared with addition-type flame retardants.^14^

Melamine is often utilized in polyurethane foams because of its stable triazine ring and high nitrogen content, and it can increase not only the fire retardancy of the polyurethane foam, but also the smoke suppression during foam combustion.^15,16^ Nevertheless, a high melamine loading can lead to a reduction of the mechanical strength of RPUFs and an increase of the viscosity of the foaming solution. Recently, Liu *et al.* and Wang *et al.* developed urea–melamine–formaldehyde (UMF) and melamine–formaldehyde (MF) foams with excellent flame retardancy, respectively.^17,18^ However, their applications in RPUFs can be restricted by the high water content.

In this study, a series of ethylene glycol modified urea–melamine–formaldehyde resins (EUMFs) were synthesized from urea, melamine, paraformaldehyde and ethylene glycol, and then incorporated into RPUFs as a reactive-type liquid flame retardant. The effects of EUMFs, as well as the melamine loading in EUMFs, on the cell morphology, compressive strength, flammability, fire behavior and thermal stability of RPUFs were investigated.

## 2. Experimental

### 2.1. Materials

LY-4110 polyether polyol (hydroxyl value: 430 mg KOH per g; viscosity at 25 °C: 2500 mPa s) was purchased from Jiangsu Luyuan New Material Co., Ltd., (Jiangsu, China). LCN-403 polyether polyol (hydroxyl value: 730–780 mg KOH per g; viscosity at 25 °C: 20 000–50 000 mPa s) was purchased from Shandong Lianchuang Energy Saving New Materials Co., Ltd., (Shandong, China). Silicone surfactant (AK-8805) was purchased from Jiangsu Meiside Chemical Co., Ltd., (Jiangsu, China). Polyarylpolymethyleneisocyanate (PAPI) (NCO%: 34.0–35.0; average functionality: 2.8, viscosity at 25 °C: 200 mPa s) was purchased from Wanhua Chemical Group Co., Ltd., (Hubei, China). Ethylene glycol, paraformaldehyde, melamine, urea, *N*,*N*-dimethylcyclohexylamine (PC-8), tris(2-hydroxyethyl)amine (TEOA), diethanolamine (DEA) and ammonium chloride (NH_4_Cl) were purchased from Sinopharm Chemical Reagent Co., Ltd., (Shanghai, China).

### 2.2. Synthesis of EUMFs

**Scheme 1:**
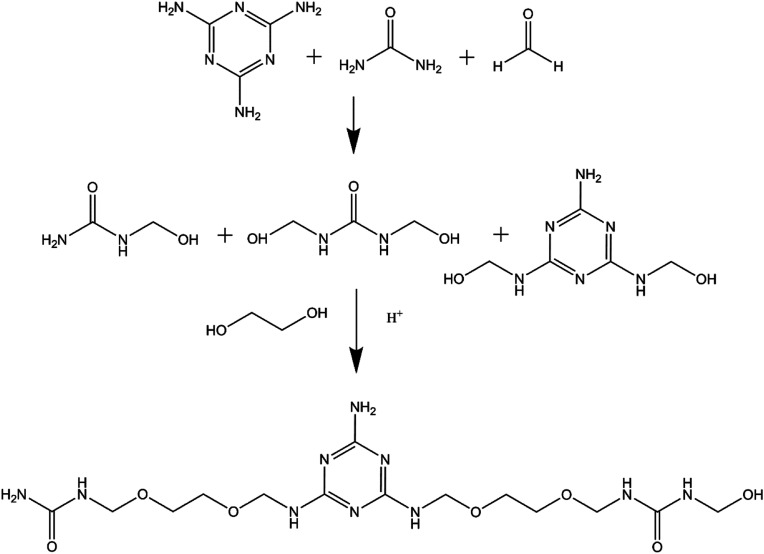
Synthesis of EUMFs.

EUMFs were synthesized using a neutral–acid–base procedure, as shown in Scheme 1, and their formulations are shown in Table 1. In brief, an appropriate amount of paraformaldehyde, ethylene glycol, urea and melamine were fed into a 1000 mL four-necked round-bottomed flask equipped with a thermometer, a mechanical stirrer and a reflux condenser, and the mixture was heated to 90 °C and then kept at that temperature for 30 min. The pH of the mixture was adjusted to 4.5–5.0 with NH_4_Cl. The degree of condensation was measured every 5 min by the turbidity point method, and one drop of resin was dispersed in 100 mL of water.^19^ When the solution became cloudy, the pH of the mixture was adjusted to 8.5–9.0 with triethanolamine. After that, the system was cooled to 60 °C and accessional urea was added, and then the resin was allowed to mature at 60 °C for 60 min. Finally, the resultant resin, a white opaque liquid, was obtained for future use. It is noted that the NH_4_Cl and triethanolamine present in the EUMF resins may have a negligible effect on the properties of the resultant products due to their small quantity and neutralization. The hydroxyl values of EUMFs were measured according to GB/T 12008.3-2009 (The Light Industry Standard of People's Republic of China), and the average of two samples was recorded.

**Table 1:** The formulations and hydroxyl values of EUMFs

		Samples			
		EUMF-1	EUMF-2	EUMF-3	EUMF-4
Composition	Urea (g)	171.0	162.0	153.0	144.0
	Melamine (g)	18.9	37.8	56.7	75.6
	Ethylene glycol (g)	31.0	31.0	31.0	31.0
	Paraformaldehyde (g)	63.0	63.0	63.0	63.0
Hydroxyl value (mg KOH per g)		460	456	446	440

### 2.3. Preparation of RPUFs

RPUFs were prepared by free-foaming according to the formulations listed in Table 2. Specifically, a fixed amount of polyols and EUMFs were mixed and stirred at a speed of 200 rpm for at least half an hour, and then the catalyst, surface active agent, and blowing agent were added and stirred for 1 min using a high speed mechanical stirrer. PAPI was added and stirred for 15 s at a speed of 2000 rpm, and then poured into an open mold for free foaming. The foams were placed in an oven for polymerization reaction at 70 °C for 24 h, and then samples were cut for characterization.

**Table 2:** The formulations of RPUFs

		Samples				
		RPUF-0	RPUF-1	RPUF-2	RPUF-3	RPUF-4
Composition	EUMF-1 (phr)	—	100	—	—	—
	EUMF-2 (phr)	—	—	100	—	—
	EUMF-3 (phr)	—	—	—	100	—
	EUMF-4 (phr)	—	—	—	—	100
	LY-4110 (phr)	135.0	35.0	35.0	35.0	35.0
	LCN-403 (phr)	60.0	60.0	60.0	60.0	60.0
	AK-8805 (phr)	1.4	1.4	1.4	1.4	1.4
	PC-8 (phr)	1.8	1.8	1.8	1.8	1.8
	PAPI (phr)	255.0	255.0	255.0	255.0	255.0
	Water (phr)	1.8	1.6	1.6	1.6	1.6
NCO/OH ratio		1.02	1.01	1.01	1.02	1.02

### 2.4. Characterization

#### 2.4.1. Molecular structure

The molecular structures of urea, melamine, EUMF-1, and EUMF-4 were characterized by Fourier transform infrared (FT-IR) and ^13^C NMR spectroscopy. The FT-IR spectra of the samples were recorded on a Nicolet 6700 spectrometer in the range of 400–4000 cm^−1^ at a scan number of 32 and a resolution of 4 cm^−1^, and the ^13^C NMR spectra were recorded on a Bruker Avance III 400 M NMR spectrometer (Buchi, Switzerland) with a relaxation delay of 5 s. The samples were prepared by dissolving approximately 50–60 mg of product in 0.5 mL of deuterated dimethyl sulfoxide (DMSO-D6).

#### 2.4.2. Density measurement

The densities of RPUF samples of 30 × 30 × 30 mm^3^ (length × width × thickness) were measured according to GB/T 6343-2009 (Standardization Administration of the People's Republic of China, the same below), and the average of five samples was recorded.

#### 2.4.3. Compressive strength measurement

The compressive strengths of RPUF samples of 50 × 50 × 50 mm^3^ (length × width × thickness) were measured with a SANS CMT-4304 universal mechanical tester according to GB/T 8813-2008 at a crosshead speed of 2.5 mm min^−1^.

#### 2.4.4. Limiting oxygen index (LOI) measurement

The LOIs of RPUF samples of 127 × 10 × 10 mm^3^ (length × width × thickness) were measured at room temperature according to GB/T 2406.2-2009 using a JF-3 oxygen index instrument.

#### 2.4.5. Thermogravimetric analysis (TGA)

TGA was performed on a NETZSCH STA 409 PC instrument at a heating rate of 10 °C min^−1^, and the foams were heated from 50 °C to 700 °C at a nitrogen flow of 50 mL min^−1^.

#### 2.4.6. Cone calorimeter testing (CCT)

CCT was performed using a FTT2000 cone calorimeter instrument according to ISO 5660-1. Each sample of 97 × 97 × 20 mm^3^ (length × width × thickness) was wrapped in aluminum foil and exposed horizontally to an external heat flux of 35 kW m^−2^. At least three samples were tested in each experiment.

#### 2.4.7. Scanning electron microscopy (SEM)

The morphologies of foams and foam residuals after CCT were characterized by a Hitachi S-4800 scanning electron microscope at an accelerating voltage of 15 kV. The residual surfaces were coated with a thin gold layer before SEM observation.

## 3. Results and discussion

### 3.1. FT-IR spectra of EUMFs

**Fig. 1:**
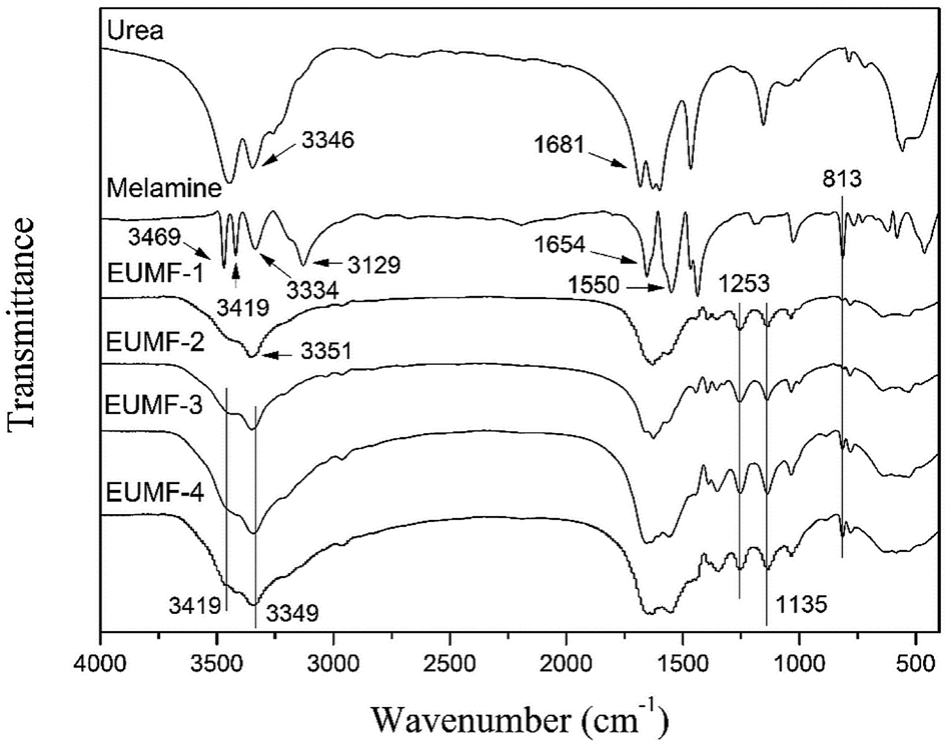
FTIR spectra of urea, melamine, and EUMFs.

The FT-IR spectra of urea, melamine, and four EUMF samples (EUMF-1 to EUMF-4) are shown in Fig. 1. In the FT-IR spectra of urea, the absorption peaks at 3346 and 1681 cm^−1^ can be assigned to N–H and C=O stretching vibration, respectively. In the FT-IR spectra of melamine, the absorption peaks at 3469, 3419, 3334 and 3129 cm^−1^ are assigned to –NH_2_ stretching vibration; whereas those at 1654, 1550 and 813 cm^−1^ are assigned to the triazine ring, respectively.^20^ In the FT-IR spectra of EUMFs, the peaks at 1135 and 1253 cm^−1^ are attributed to the absorption of C–O–C and –CH_2_ of –CH_2_–O–CH_2_– groups, respectively. The absorption peak at 3351 cm^−1^ in the FT-IR spectra of EUMF-1 is assigned to –NH–, and those at 3419 and 3349 cm^−1^ in the FT-IR spectra of EUMF-2, EUMF-3, and EUMF-4 are assigned to –NH_2_, respectively.^21,22^

### 3.2. ^13^C NMR analysis of EUMF-2

**Fig. 2:**
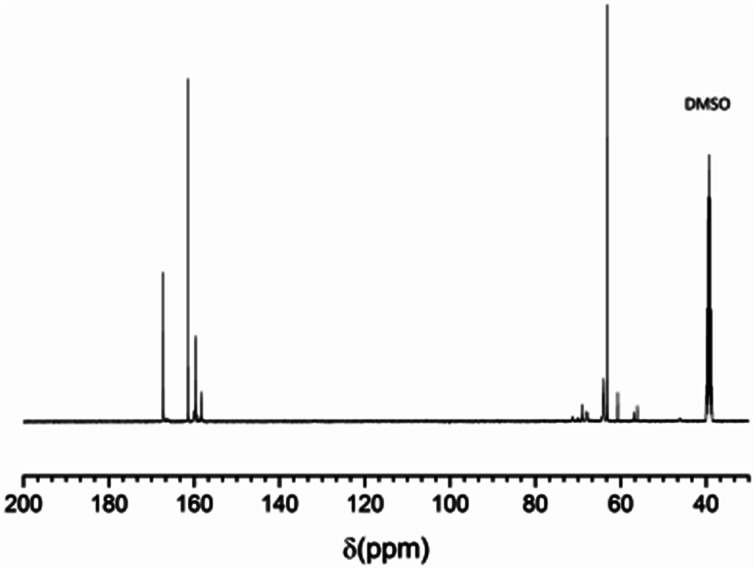
^13^C NMR spectrum of EUMF-2.

All EUMF samples show similar ^13^C NMR spectra. As an example, Fig. 2 shows the ^13^C NMR spectrum of EUMF-2, and its peak assignments are shown in Table 3. The peaks at 46.0, 56.2–57.0, and 60.7 ppm in the ^13^C NMR spectrum of EUMF-2 are attributed to the signals of methylene, and those at 63.1–63.9 ppm are attributed to the signals of methylol. The ^13^C absorption in the dimethylene ether group is observed at 68.0–69.0 and 70.1–71.3 ppm.^23^ In addition, the ^13^C absorption in urea is observed at 161.3 ppm; while that in mono-substituted and di, tri-substituted urea appears at 158.3 and 159.3–160.0 ppm, respectively. The peaks at 166.3–167.2 ppm can be attributed to the signals of substituted triazine.^19,24^ All these results indicate the occurrence of amine–aldehyde condensation reaction in the system.

**Table 3:** ^13^C NMR assignments of EUMF-2

Substance and structure		Chemical shift (ppm)
Methylene	–NH–CH_2_–NH–	46.0
	–N(CH_2_–)–CH_2_–NH–	56.2–57.0
	–N(CH_2_–)–CH_2_–N(CH_2_–)–	60.7
Methylol	–NH–CH_2_OH	63.1–63.9
Dimethylene ether	–NH–CH_2_–O–CH_2_–NH–	68.0–69.0
	–N(CH_2_–)–CH_2_–O–CH_2_–NH–	70.1–71.3
Urea	NH_2_CONH_2_	161.3
Mono-substituted urea	–NHCONH_2_	159.3–160.0
Di, tri-substituted urea	=NCONH–, –NHCONH–	158.3
Substituted triazine	=CNHCH_2_OH	166.3–167.2

### 3.3. Cell morphology, apparent density and physical–mechanical properties of RPUFs

**Fig. 3:**
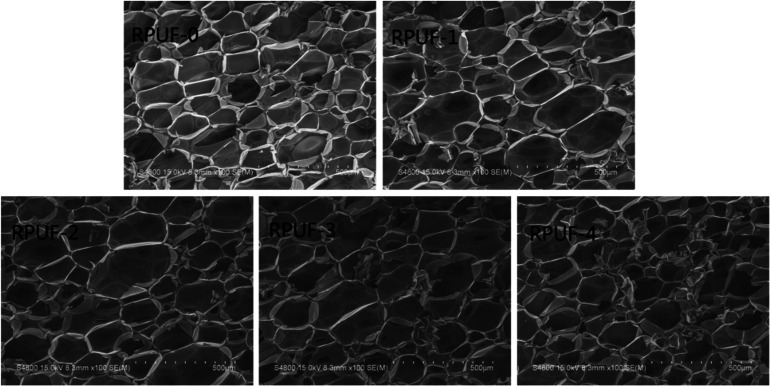
SEM images of RPUFs.

The effect of EUMFs on the microstructure of RPUFs was examined by SEM. The cells of RPUF-0 are quadrilateral or hexagon shaped with a uniform diameter (Fig. 3), while those of RPUFs become less regular in shape with a larger and nonuniform diameter. This can be attributed to the branched structure of EUMFs, which can have a significant effect on the mechanical properties of RPUFs.

Given the remarkable effects of foam apparent density on the mechanical properties, flammability, and combustion performance, the apparent density is controlled at about 50 kg m^−3^.^25^ The foams were compressed to 10% of their original thickness at a crosshead speed of 2.5 mm min^−1^, and their physical and mechanical properties were determined, as shown in Table 4. The maximum compressive strength is observed in RPUF-0, indicating that the addition of EUMFs can impair the compressive strength of RPUFs, which is likely due to the destruction of cell structure. However, as the melamine loading in EUMFs increases from 18.9 g to 75.6 g, the compressive strength of RPUF-4 is increased by 24.62% compared with that of RPUF-1, which is mainly due to the high stability of triazine ring that can improve the stiffness of RPUFs.^17^

**Table 4:** Apparent density and compressive strength of RPUFs

Samples	Density (kg m^−3^)	Compressive strength (kPa)
RPUF-0	49.36 ± 1.08	256.9 ± 14.7
RPUF-1	51.26 ± 1.29	186.8 ± 12.6
RPUF-2	50.96 ± 1.78	199.7 ± 14.5
RPUF-3	51.70 ± 1.67	223.6 ± 8.8
RPUF-4	50.04 ± 0.94	232.4 ± 16.3

### 3.4. Flammability of RPUFs

The flammability of RPUFs was characterized by LOI. As shown in Table 5, the LOI value of RPUF-0 is 18.1%, and it is increased to about 24% with the incorporation of EUMFs. The improved flame retardancy of RPUFs can be attributed to the release of non-flammable gases and the increase of char yield during the combustion of RPUFs.^17^ Nevertheless, increasing the melamine loading in EUMFs has a negligible effect on the LOI values of RPUFs.

**Table 5:** The LOI values of RPUFs

Samples	LOI (%)
RPUF-0	18.1
RPUF-1	24.2
RPUF-2	24.3
RPUF-3	24.4
RPUF-4	24.4

### 3.5. Fire behaviors of RPUFs

**Fig. 4:**
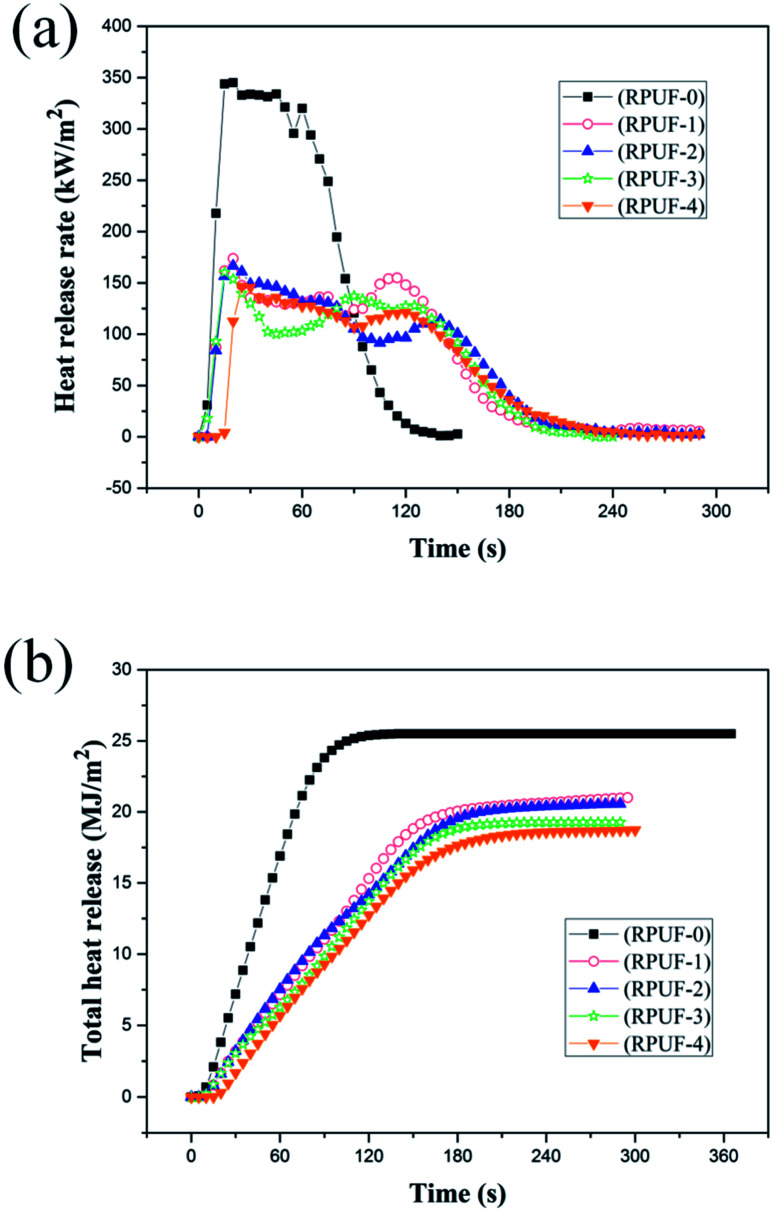
The HRR curves (a) and THR curve (b) of RPUFs.

**Fig. 5:**
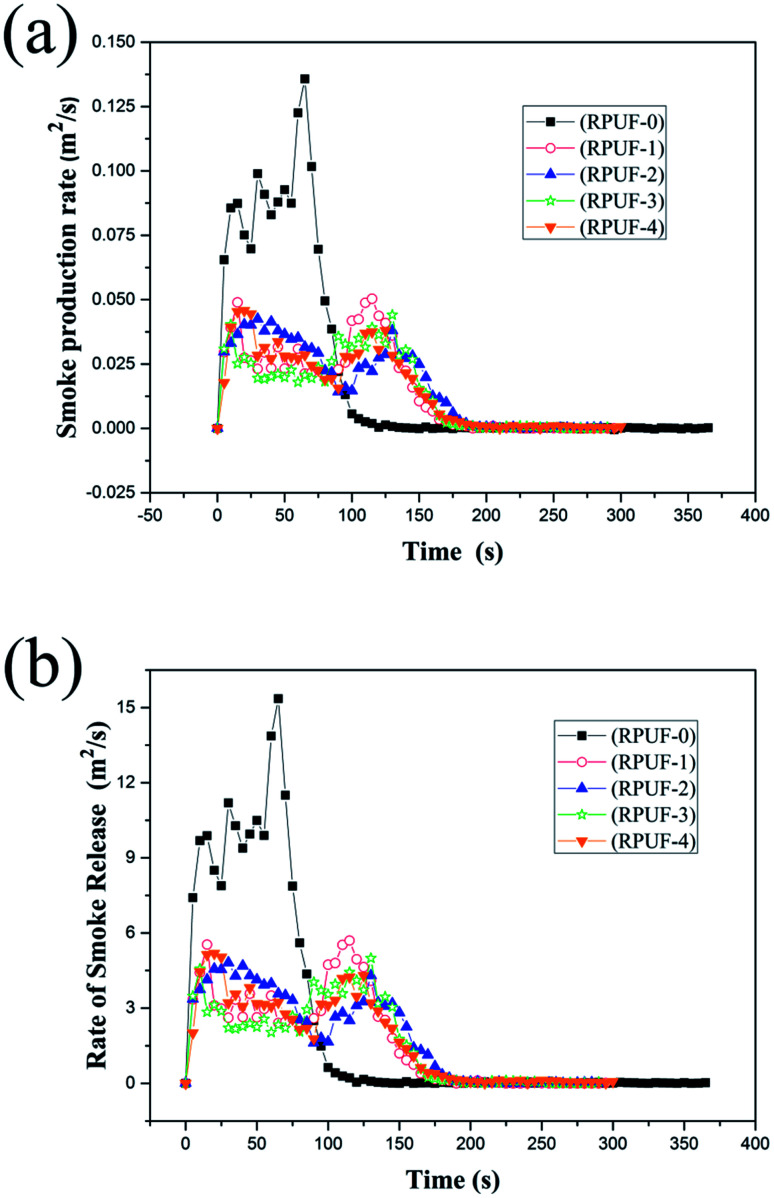
The SPR curves (a) and RSR curves (b) of RPUFs.

The fire behaviors of RPUFs were characterized by CCT at a heat flux of 35 kW m^−2^, as shown in Fig. 4 and 5 and Table 6. In this study, the parameters of interest include the time to ignition (TTI), heat release rate (HRR), peak heat release rate (PHRR) and total heat release (THR). As shown in Table 6, the TTIs of both flame retardant foams and original foams are very short due to the cell structure of RPUFs. HRR is considered as a measure of fire intensity. As shown in Fig. 4(a), there are two peaks in the HRR curve of RPUF-0. The first peak is caused by the formation of a char layer on the surface of RPUF-0 during combustion. The inner polymer is exposed to the flame due to the pyrolysis of the char layer, resulting in the formation of a second HRR peak at about 60 s followed by a sudden reduction.^26^ The addition of EUMFs results in a decrease in PHRRs of RPUFs to 173.7, 166.5, 160.2, and 146.4 kW m^−2^, respectively, and a delay of the second HRR peak to about 120 s. It can also be observed that melamine plays an important role in reducing the PHRRs of RPUFs. The PHRRs of RPUFs gradually decrease with increasing melamine loading in EUMFs, which is attributed to the formation of melam during the thermal condensation of melamine.^27^ The THRs of RPUFs are decreased by 17.6, 19.2, 24.3, and 26.7% compared with that of RPUF-0, respectively, indicating that the incorporation of EUMFs can effectively reduce the combustion intensity of RPUFs. This may be because (1) EUMFs decompose at a lower temperature than polyurethane foam and release non-flammable gases, such as NH_3_, HNCO, and HCN, which can reduce the concentration of flammable fragments; and (2) the incorporation of EUMFs enables the char layer to have better anti-oxidation properties, so that the HRR is lower than that of original foam.

**Table 6:** Flammability test and smoke emission behaviors of RPUFs

Samples	RPUF-0	RPUF-1	RPUF-2	RPUF-3	RPUF-4
TTI (s)	2	3	3	3	3
PHRR (kW m^−2^)	345.2	173.7	166.5	160.2	146.4
THR (MJ m^−2^)	25.5	21.0	20.6	19.3	18.7
PSPR^a^ (m^2^ s^−1^)	0.136	0.050	0.043	0.044	0.046
TSP (m^2^)	7.5	4.6	4.9	4.3	4.6
PRSR^b^ (m^2^ s^−1^)	15.36	5.70	4.81	4.99	5.17
TSR (m^2^ m^−2^)	849.8	519.7	548.8	487.0	518.6
CO (kg kg^−1^)	0.18	0.08	0.07	0.06	0.08
CO_2_ (kg kg^−1^)	1.93	1.35	1.45	1.37	1.38
CO/CO_2_ weight ratio	0.093	0.059	0.048	0.044	0.058

a PSPR is the peak smoke production rate.

b PRSR is the peak smoke release rate.

The smoke emission behaviors of RPUFs were characterized by the smoke production rate (SPR), total smoke production (TSP), rate of smoke release (RSR), total smoke release (TSR), and weight ratio of CO to CO_2_. As shown in Fig. 5, the SPRs and RSRs of RPUFs are quite different from those of RPUF-0. The SPR and RSR curves of RPUF-0 show a sharp and strong peak at about 65 s, while those of RPUFs show two peaks of similar strength. RPUF-3 shows the best smoke suppression performance. Table 6 shows that the SPR and TSP of RPUF-3 are reduced by about 67.6 and 42.7% compared with that of RPUF-0, respectively. This is probably due to the reaction between melamine and aromatic hydrocarbon, which is the primary source of smoke during combustion.^15,28^ However, the release of the smoke can be restricted by the compact nitrogen-rich carbon layer formed in the initial stage of combustion. Besides, as shown in Table 6, the PSPR, TSP, PRSR, TSP, and CO/CO_2_ weight ratio show no obvious decrease. The TGA results of RPUFs show that the initial degradation temperature of RPUF-4 is lower than that of RPUF-3, indicating that RPUF-4 is degraded earlier than RPUF-3. As a consequence, melamine is released earlier in RPUF-4, resulting in a decrease in the reaction between melamine and aromatic hydrocarbon. However, the difference is not so obvious.

The CO yield and CO/CO_2_ weight ratio of RPUFs are important factors for evaluating the anti-fire performance.^29^ As shown in Table 6, the CO/CO_2_ weight ratio of RPUF-3 is reduced by 52.7% compared with that of RPUF-0, which is mainly attributed to the compact char layer. The more the decomposed fragments are reserved in the residue, the less the fragments are burned in fire and consequently the less the CO is released.

### 3.6. Thermal stability of RPUFs

**Fig. 6:**
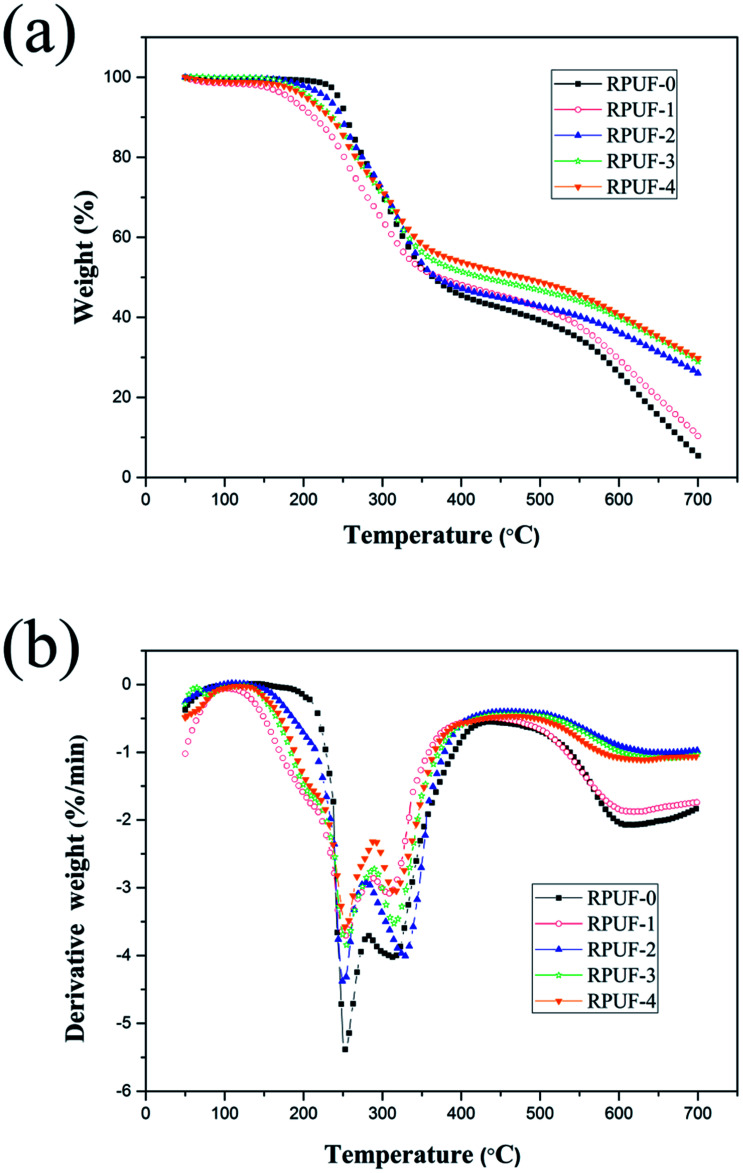
TGA (a) and DTG (b) curves of RPUFs.

TGA is widely used to evaluate the thermo-oxidative degradation behaviors of different materials.^26^ TGA and derivative thermogravimetric analysis (DTG) curves of all foams under nitrogen atmosphere are shown in Fig. 6, and the data is shown in Table 7. As shown in Fig. 6, all RPUFs are decomposed in three stages. The first decomposition stage of RPUF-0 begins at about 243 °C due to the scission of C–O bonds in the urethane group, resulting in the formation of isocyanates and polyols. As the pyrolyzation proceeds, imidodicarbonic diamide is released in the self-reaction of partial isocyanates, which is accompanied by the volatilization of carbon dioxides, alcohols, amines, aldehydes and carbon monoxides.^30^ The second decomposition stage is observed at 282–430 °C, which corresponds to the degradation of imidodicarbonic diamide and substituted urea generated in the reaction between isocyanates and polyols or water.^31^ The third decomposition stage starts at about 430 °C due to further decomposition of char residue.

**Table 7:** TGA data of RPUFs

Samples	*T*_initial_ a (°C)	Stage 1		Stage 2		Stage 3		Char residue at 700 °C (%)
		*T*_max_ b (°C)	*W*_1_ c (%)	*T*_max_ b (°C)	*W*_2_ c (%)	*T*_max_ b (°C)	*W*_3_ c (%)	
RPUF-0	243	252	90.7	312	64.5	617	22.3	5.4
RPUF-1	179	251	80.1	308	61.6	618	25.8	10.3
RPUF-2	216	255	83.7	315	67.2	630	35.4	26.1
RPUF-3	207	251	88.2	329	60.9	659	34.6	29.0
RPUF-4	202	254	84.3	314	66.0	664	33.0	29.8

a *T*_initial_ is the initial degradation temperature (temperature at 5.0% weight loss).

b *T*_max_ is the maximum-rate degradation temperature.

c *W* is the weight remaining percentage at the maximum-rate degradation temperature.

The initial decomposition temperature of RPUFs is slightly lower than that of RPUF-0, because EUMFs can be decomposed more easily. For RPUFs, the EUMFs can decompose into NH_3_, HNCO, HCN, melamine and some stable intermediates at about 170 °C. Melem is a direct condensation product of melamine at high temperatures, which can promote the formation of a dense char layer on the surface of the burning material.^27^ As a consequence, the residue char of RPUFs is significantly increased. For instance, the char residue of RPUF-4 is increased to 29.8%. The dense char layer plays a crucial role in isolating the polymer matrix from heat. As shown in Fig. 6(b), the maximum-rate degradation temperature of the second and third decomposition stages of RPUFs is similar to that of RPUF-0, and the addition of EUMFs results in a decreases of decomposition rate.

### 3.7. SEM images of residues after CCT

**Fig. 7:**
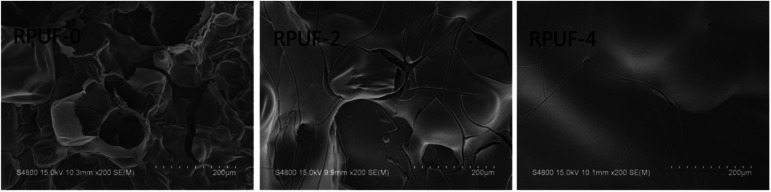
SEM images of RPUFs residues after CCT.

Fig. 7 shows the SEM images of RPUF-0, RPUF-2, and RPUF-4 after CCT. A thin and holey carbonaceous layer is formed in RPUF-0, making it difficult to inhibit heat and mass transfer. Compared with RPUF-0, the char residues of RPUFs become dense, which contributes significantly to the good flame retardancy of RPUFs as it can isolate the inner polymer from heat and oxygen.^26^

## 4. Conclusions

In this study, a series of EUMFs were synthesized by amine–aldehyde condensation and aldolization, and then incorporated into RPUFs to prepare reactive-type flame retardants. The incorporation of EUMFs can adversely affect cell morphology and compressive strength of RPUFs, but result in an increase in the LOI value of RPUFs to about 24%. Nevertheless, the melamine loading in EUMFs has a negligible effect on the LOI value of RPUFs. The CCT results reveal that RPUFs exhibit good fire resistance and smoke suppression. In particular, the TSP, TSR and CO/CO_2_ weight ratio of RPUFs decrease significantly with the incorporation of EUMFs, and that of RPUF-3 are decreased by about 42.7%, 42.7% and 52.7% in comparison with that of RPUF-0, respectively. The dense char layer contributes significantly to the good fire resistance and smoke suppression of RPUFs.

## Conflicts of interest

There are no conflicts to declare.

## Supplementary Material
